# Immunosenescence: Shaping the Hematological Narrative in the Era of Aging

**DOI:** 10.14336/AD.2025.0741

**Published:** 2026-09-06

**Authors:** Yajie Wu, Ji Luo, Jiaoya Lin, Shuai Zheng, Yingmiao Wu, Yifei Gao, Jiao Chen, Feifei Che, Ling Zhong

**Affiliations:** ^1^The Genetic Diseases Key Laboratory of Sichuan Province, The Department of Medical Genetics, The Department of Laboratory Medicine, Sichuan Academy of Medical Sciences & Sichuan Provincial People's Hospital, School of Medicine, University of Electronic Science and Technology of China, Chengdu, China.; ^2^School of Basic Medical Sciences, Southwest Medical University, Luzhou, Sichuan, 646000, China.; ^3^Department of Blood Transfusion, Sichuan Academy of Medical Sciences & Sichuan Provincial People's Hospital, Chengdu, Sichuan, 610072, China.; ^4^Department of Hematology, Sichuan Provincial People's Hospital, University of Electronic Science and Technology of China, Chengdu, Sichuan, 610072, China.

**Keywords:** Immunosenescence, Hematological Diseases, Therapeutic Strategies

## Abstract

Aging has emerged as a critical focus in understanding human diseases. Among many factors in aging, immunosenescence stands out as a significant focus. Immunosenescence is the process of physiological and functional deterioration of the immune system, which consists of organs, cells, immune components and regulatory networks. In hematologic diseases, immunosenescence could affect the bone marrow (BM) microenvironment, resulting in reduced function of hematopoietic stem and progenitor cells, and interfering with normal hematopoiesis. However, how the nature and function of immune cells change during immunosenescence and the effects have not yet been summarized and explored. Therefore, this review reveals the dual role of immunosenescence in increased disease risk and reduced therapeutic efficacy by providing an overview of the characteristics of immunosenescence, the interaction with the mechanisms of development of hematologic diseases, and the impact of immunosenescence on the function of individual immune cells during the diseases. It includes separate descriptions of various immune cells (innate and adaptive immune response cells) involved in immunosenescence, which covers changes in their cell numbers, subpopulations, receptors, cytokine levels, and other aspects. Additionally, it provides an in-depth analysis of how these changes influence the progression of hematologic diseases (both neoplastic and non-neoplastic hematological diseases). Additionally, we explore the impact of senescence on therapeutic strategies and propose treatment programs for elderly patients. With this review, we endeavor to further disclose the mystery of how immunosenescence impacts the onset and progression of hematologic disorders, aiming to offer a scientific footing for devising novel therapeutic approaches for elderly patients and to enhance their prognosis.

## 1. Introduction

With the continuous advancement of modern medical standards, the average human lifespan has been extended [1], leading to an aging population. The elderly are not only at a higher risk of developing age-related diseases, including cancer, but also face higher failure rates in immunotherapy and increased recurrence rates post-treatment [2]. Among the myriad of factors affecting the health and prognosis of the elderly, aging stands out as a key player. A significant branch of aging is immunosenescence, which encompasses the aging of various immune systems and immune cells, primarily characterized by increased incidence of infections, increased susceptibility to diseases, and impaired response to new antigens [3, 4].

Immunosenescence is one of the important factors contributing to the development of many diseases, which can affect various aspects of the immune system and impact the progression and prognosis of diseases from both increasing the risk and reducing the effectiveness of treatment perspectives. In hematologic malignancies, senescence-related immune dysfunction promotes tumor growth. Conversely, disease progression accelerates aging processes [5]. Immunosenescence can lead to an increase in immunosuppressive factors (such as PD-L1, TGF-β, etc.) in the tumor microenvironment, inhibiting the function of immune cells, thereby helping tumors evade immune clearance and enhancing the risk of cancer. The increased low-grade inflammatory state accompanying immunosenescence may promote tumor growth. The imbalance in the immune system may enhance suppressive immune functions, reducing the effectiveness of immunotherapy against tumors. The tumor escape mechanism may be strengthened with age, making the elderly less responsive to treatments like immune checkpoint inhibitors [6, 7] (Fig. 1). Recent research emphasizes that the upregulation of methylmalonic acid in the serum of the elderly induces the expression of Sox4, triggering the ability to reprogram transcription, which can make cancer cells more aggressive. However, aging has also been found to improve cancer prognosis, possibly because host aging factors hinder the growth and spread of aggressive tumors [8].

**Figure 1.**
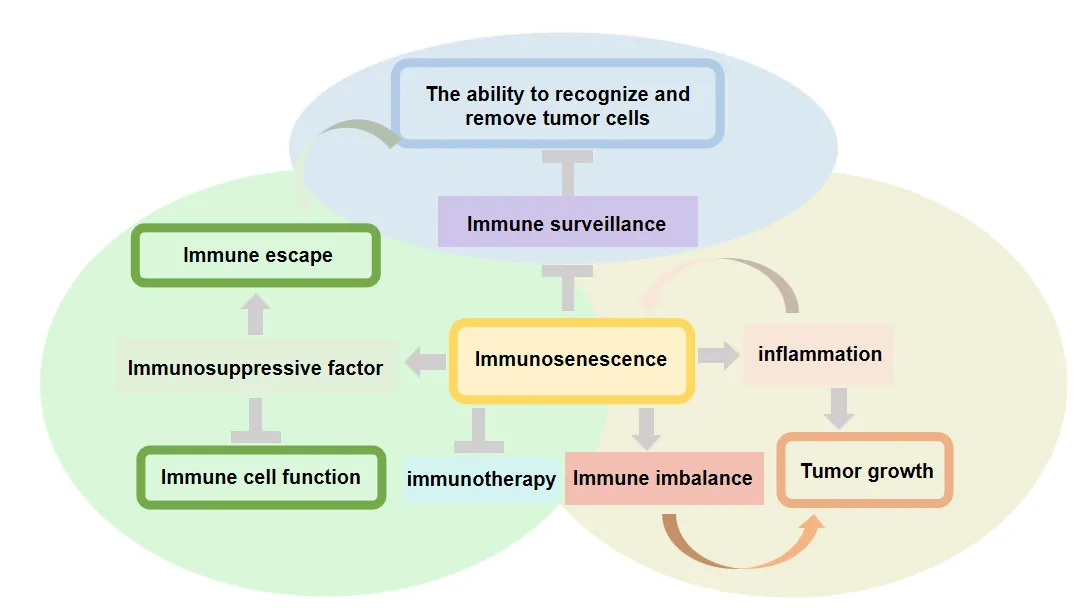
Effects of immunosenescence: immunosenescence effects immune surveillance, the balance of the immune system, the level of immunosuppressive factors, the effectiveness of immunotherapy, and the state of inflammation, which in turn can exacerbate the process of immunosenescence.

Given the relationship between immunosenescence and hematological diseases, research in this area may pave the way for breakthroughs in the treatment of hematological system diseases, thereby extending human lifespan. This review starts with the characteristics and mechanisms of immunosenescence, explores the changes in various immune cells under immunosenescence and their relationship with the pathogenesis of hematological diseases, and focuses on the impact of immunosenescence on the occurrence and progression of hematological diseases, as well as therapeutic strategies. The synthesis aims to deepen understanding of modulating immunosenescence to ameliorate its effects on blood disorders.

## 2. Immunosenescence

Immunosenescence refers to the degeneration and remodeling of immune organ structures, as well as the decline in innate and adaptive immune functions that occur with aging. These changes result in diminished vaccine efficacy, increased susceptibility to infections, and a higher risk of age-related diseases and malignancies. Immunosenescence is driven by both external factors (e.g., UV exposure, smoking, pollution) and intrinsic factors (e.g., aging, genetics, chronic diseases) [8, 9]. These factors interact with each other and promote the process of immunosenescence (Fig. 2)

The immune system consists of multiple key components: immune organs, immune cells, and immune active substances, all of which are closely related to immunosenescence. The thymus, as one of the most important immune organs, undergoes degeneration during the process of immunosenescence, a process considered a significant characteristic of immunosenescence [8]. The thymus plays a crucial role in human development, primarily responsible for the generation and maturation of T cells [9]. However, during the aging process, thymic function gradually declines [10], leading to a decrease in the number of newly generated naive T cells. In contrast to newly generated naive cells, memory cells have a longer lifespan. After the decline of thymic function, memory T cells previously activated by antigens progressively accumulate in the body. This has led to an imbalance between immature cells and memory cells in the immune system, which may have a significant impact on the function of the immune system [11]. In addition, the thymus is also an important hormone-secreting organ, and its functional degeneration leads to changes in hormone levels, which may cause metabolic disorders in the body and subsequently affect overall health status. Immunosenescence not only affects the structure and function of organs but also profoundly affects the internal regulatory network of immune cells. This imbalance changes the expression and regulation patterns of genes, leading to epigenetic changes. These combined factors lead to a decline in immune system function and a weakening of immune responses, which are important characteristics of the process of immunosenescence [9].

**Figure 2.**
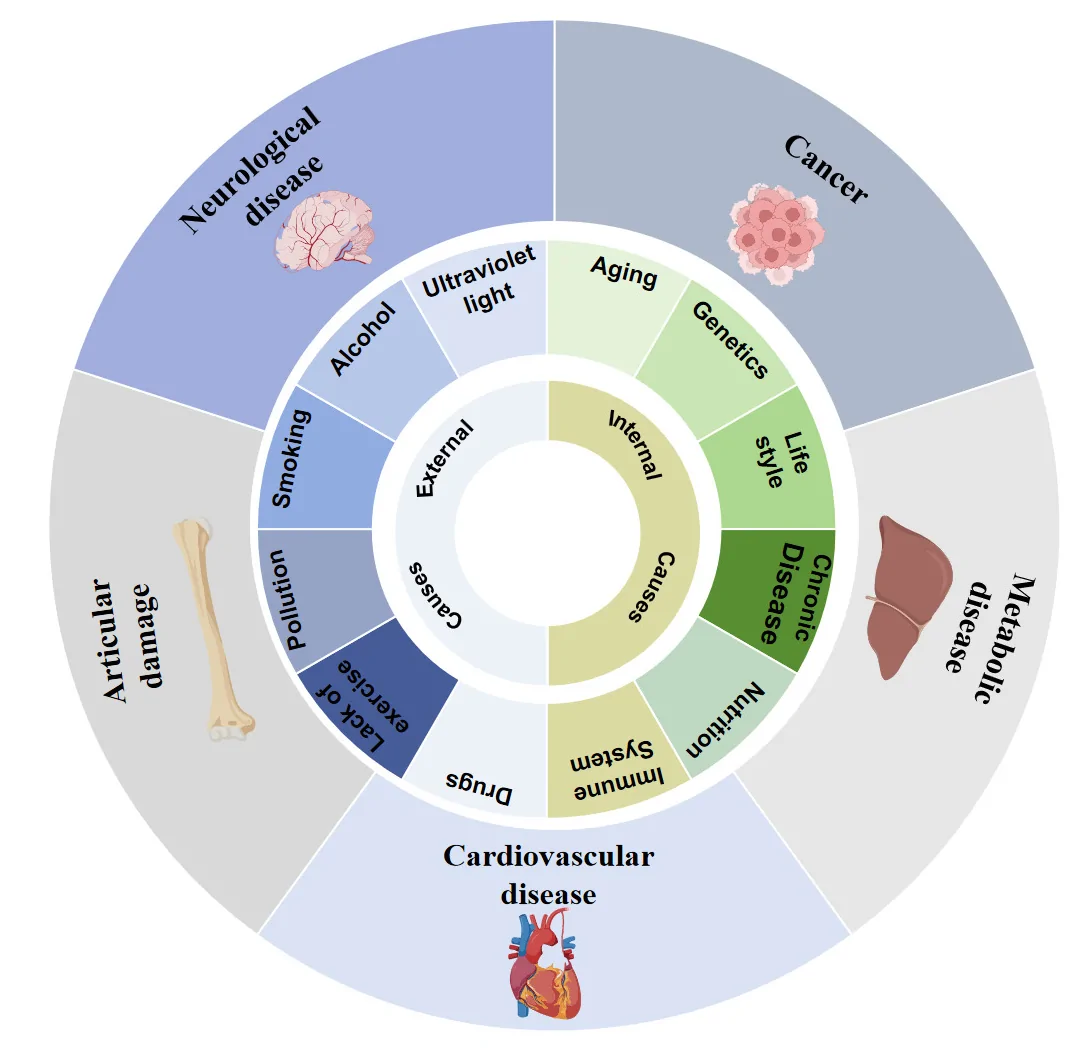
Multiple internal and external factors influence immunosenescence, which in turn is associated with a variety of diseases.

Senescence-Associated Secretory Phenotype (SASP) is a key characteristic exhibited by senescent cells, referring to the complex phenomenon of their secretion of various bioactive molecules, including pro-inflammatory cytokines, chemokines, growth factors, etc. These bioactive molecules amplify the inflammatory response through a positive feedback loop, leading to inflammatory aging [12]. Although the molecular mechanisms of immunosenescence remain unresolved, existing literature suggests that senescent T cells and inflammation may be the primary drivers of immunosenescence. A substantial body of research converges on the consensus that the antigenic burden to which an individual is exposed throughout life is associated with immunosenescence, with a disrupted T cell repertoire and chronic antigenic stimulation mediating premature aging of immune cells [13].

T cell senescence involves multiple pathways, persistent antigenic stimulation and genetic damage due to competition for scarce nutrients in the TME initiate senescence. Upregulated Tregs compete with effector T cells for glucose, triggering ATM-dependent NA damage. This activates the ERK1/2, p38, and STAT1/3 pathways, upregulates p21, p16, and p53 expression, and ultimately drives irreversible T-cell cycle exit [13-15]. Adenosine 5’monophosphate-activated protein kinase (AMPK) pathways activated by glucose deprivation could down-regulate telomerase reverse transcriptase gene binding to transforming growth factor-activated protein kinase binding protein 1 (TAB1), causing autophosphorylation of p38, which in turn initiates T-cell senescence and DNA damage [16]. Eventually, cell proliferation was blocked and autophagy was inhibited (Fig. 3).

**Figure 3.**
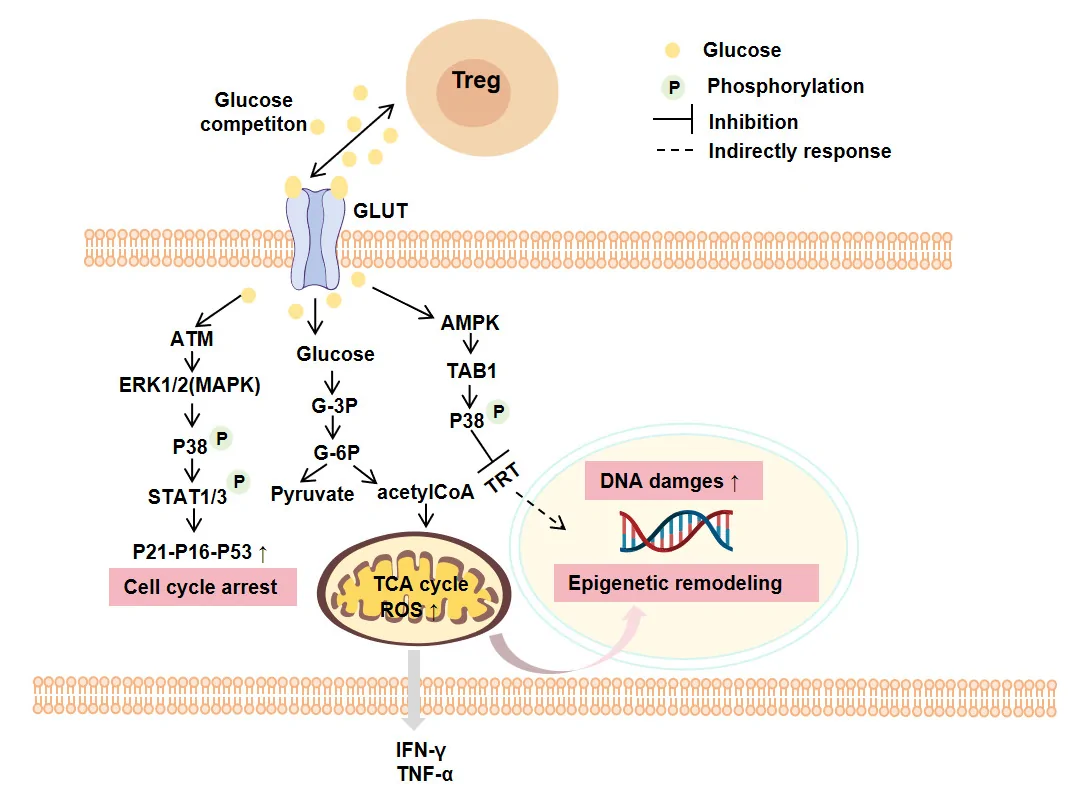
**Multiple pathways are involved in T-cell senescence: glucose competition triggers ATM-dependent DNA damage, activating ERK1/2 and p38 pathways while engaging STAT1/3, ultimately causing T cell cycle arrest and senescence**. p38 activation downregulates TERT, exacerbating DNA damage. Reduced TCR signaling further activates p38 while suppressing PI3K-AKT-mTOR signaling, resulting in impaired autophagy, mitochondrial dysfunction, and ROS accumulation in senescent T cells.

Aging leads to immunosenescence—a progressive remodeling of the immune system that dysregulates cellular immunity, impairs long-term immune responses, and critically compromises host defense against infections and inflammatory stimulation (Fig. 4).

## 3. immunosenescence of innate immune response cells

### 3.1 Natural killer (NK) Cells:Guardian of the immune system

NK cells are an indispensable part of the innate immune system, uniquely identifying and eliminating variant cells, such as tumor and virus-infected cells. Moreover, NK cells rapidly process senescent cells [17-20] and coordinate with other immune components through the production of cytokines and chemokines to monitor and eliminate cancer cells. Aging is associated with the redistribution of NK cell subsets, a decrease in the expression of activation receptors, and a reduction in cytotoxicity (Table 1).

**Figure 4.**
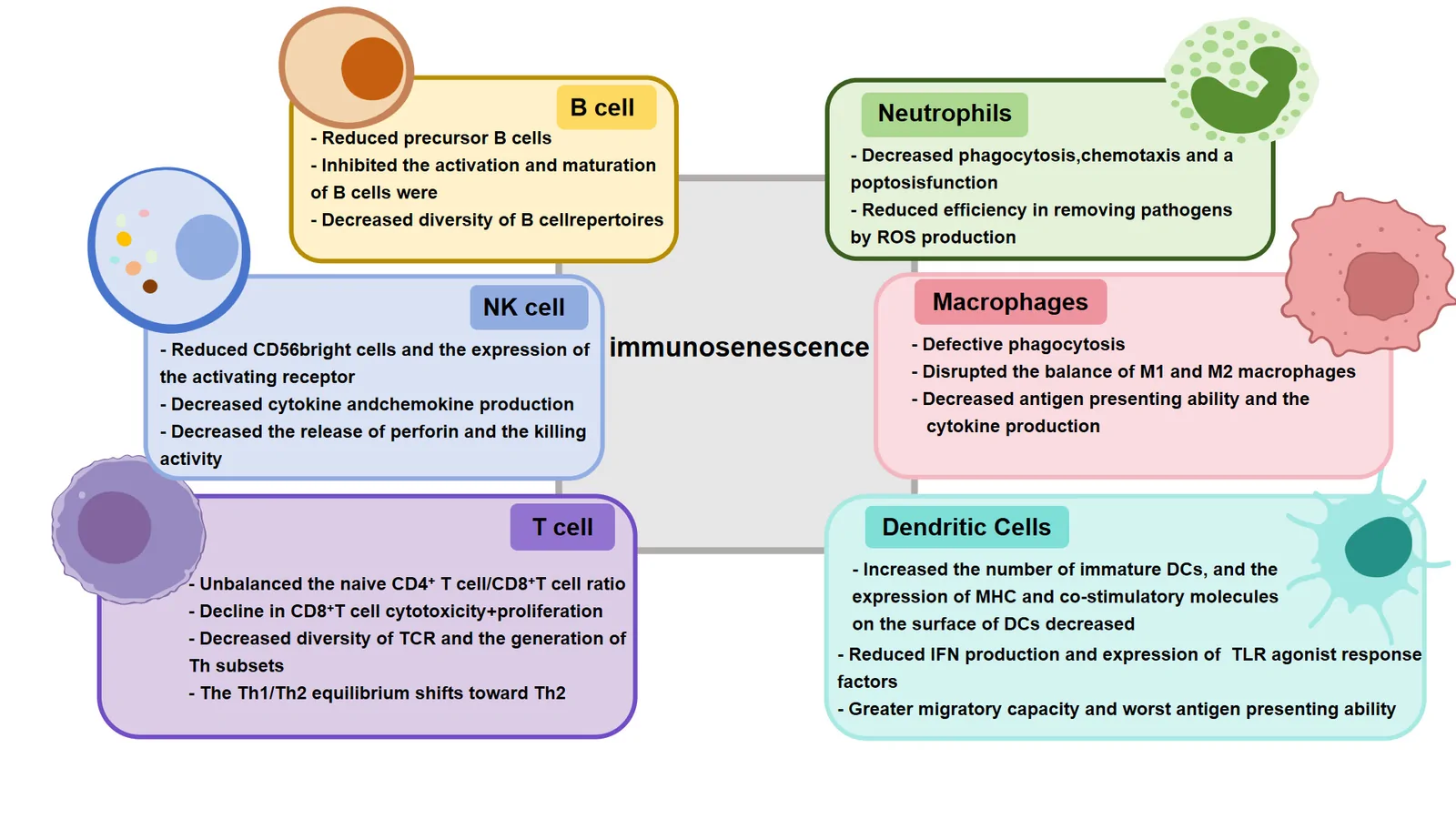
**The impact of immunosenescence on immune cells**. A brief summary of the most important age-related immune changes.

NK cells constitute 10-20% of the circulating lymphocyte pool and exhibit different subsets based on the expression density of CD56. Approximately 90% of peripheral blood NK cells are CD56dim, which are known for their high cytotoxicity but low production of cytokines and chemokines upon activation. In contrast, 10% of CD56bright NK cells excel in proliferation and produce a variety of cytokines and chemokines, albeit with minimal cytotoxicity [21]. With the advent of immunosenescence, significant changes occur in human NK cells. The proportion of the CD56bright subpopulation declines, whereas the CD56dim subpopulation is significantly elevated (up to 80%-90% of total NK cells). However, the cytotoxicity of this subpopulation is significantly attenuated. Mechanistically, the decrease in the content of key effector molecules (perforin, granzyme B, CD16, etc.) may be related to the accumulation of ROS due to aging-associated mitochondrial dysfunction, which destabilizes the lysosome and inhibits the synthesis of toxic particles. The promoter regions of perforin, CD16 and other genes are hypermethylated and transcriptionally blocked in senescent NK cells. In addition, the expression of activation receptors (NKG2D, DNAM-1, MICA/B, etc.) is down-regulated, which further reduces the ability of NK cells to recognize target cells. This paradoxical phenomenon of “increase in number and decrease in function” is essentially a kind of senescence-associated functional depletion. The increase in the ratio of CD56dim is only a quantitative compensation, but the cells have already entered into an irreversible state of senescence, and it is difficult to repair the functional damage. Moreover, increased expression of the highly differentiated NK cell marker CD57 in the elderly, expansion of CD56dimCD57+ NK cells [22-24].

**Table 1:** Effects of aging on NK cells

Cytokines	Change
**NK cell subsets**	
**CD56brightNK cells**	decreased
**CD56dimNK cells**	increased
**Function**	
**Natural cytotoxicity**	decreased
**ADCC**	decreased
**Proliferation**	decreased
**Response to cytokines**	decreased
**Cytokine and chemokine production**	decreased
**Surface markers**	
**CD57 expression on CD56dim cells**	increased
**NKG2A**	increased
**NKG2B&NKG2C**	decreased
**Functional markers**	
**IFN-γ**	decreased
**IL-8**	decreased
**TNF-α**	increased

Compared to CD57- NK cells, CD57+ NK cells exhibit enhanced cytotoxicity against target cells but reduced responsiveness to cytokines. This change is one of the signs of terminal differentiation and dysfunction of NK cells. These cells are highly differentiated, displaying a mature phenotype, with increased cytotoxic capacity, heightened sensitivity to CD16 stimulation, and decreased responsiveness to cytokines. Their diminished proliferative capacity supports the notion of a functional shift from CD56bright to CD56dim to CD56dimCD57- and then to CD56dimCD57+ NK cells during the differentiation process. This transition may be attributed to a reduction in the production of BM, precursors in elderly individuals, hematopoietic stem cells (HSCs) in aging individuals tend to differentiate into myeloid lineage rather than lymphoid lineage, resulting in a reduction in the production of CD56bright (immature subpopulation) [25] (Fig. 5). Age and persistent cytomegalovirus (CMV) infection both contribute to the phenotypic and functional changes of NK cells in the elderly. Nevertheless, NK cells continue to play a critical role in the surveillance of senescent cells (SCNs) [17, 26], and the accumulation of SCNs is a major driving factor of the SASP as age increases [18, 20].

**Figure 5.**
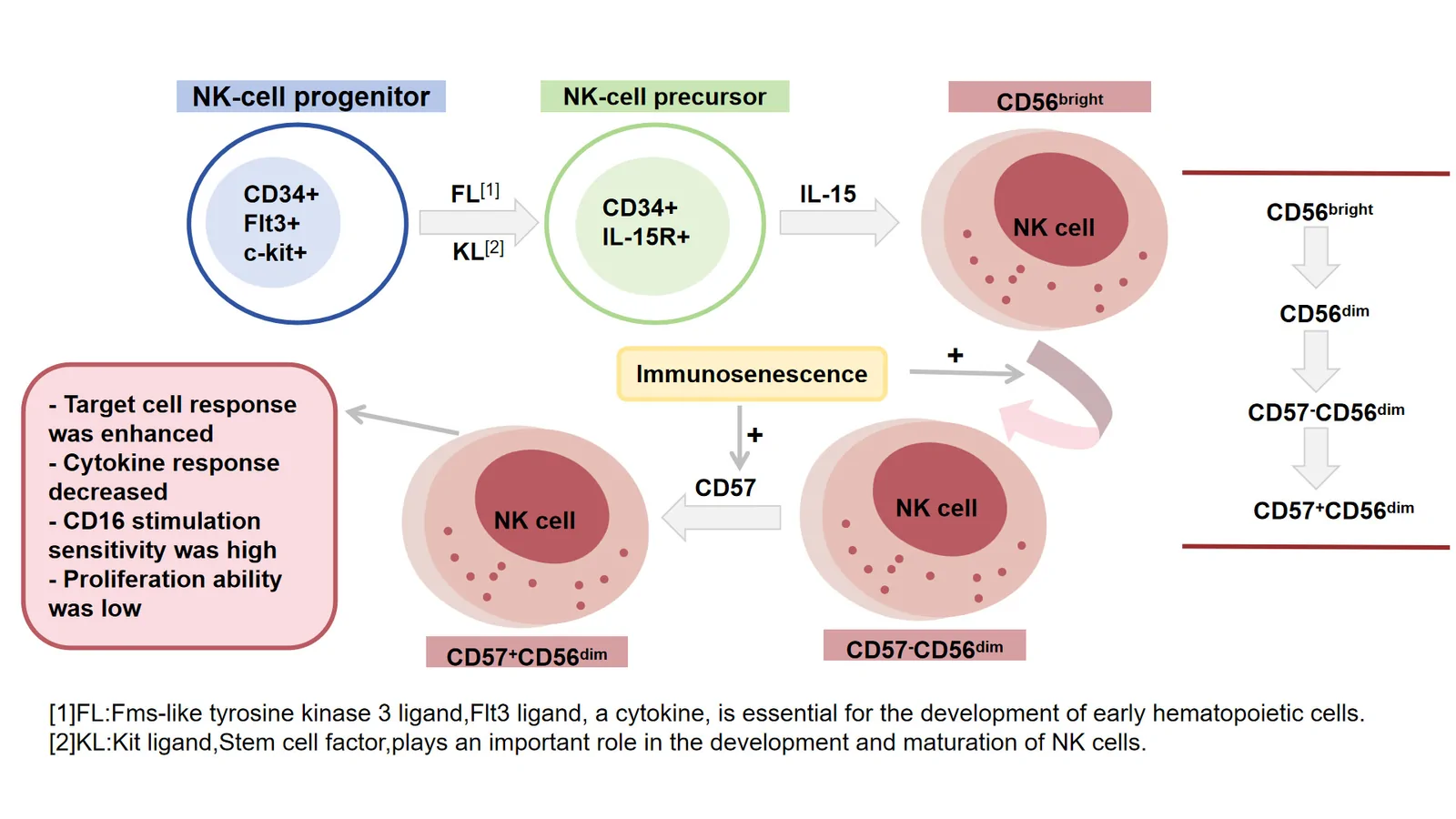
**Effects of immunosenescence on the development of NK cell subsets: immunosenescence promotes the transformation of CD56bright NK cells into CD56dim NK cells**. This process is accompanied by increased CD57 expression, leading to functional alterations in target cell response, cytokine production, and proliferative capacity.

Exhausted NK cells experience upregulation of PD-1 and KLRG1 receptors, while cytokine production is downregulated. Compared to young individuals, healthy elderly people have lower levels of IFN-γ, IL-8, and chemokines produced by NK cells both at rest and upon activation [27]. The expression of activating receptors on the surface of NK cells is decreased in the elderly. The body may attempt to maintain immune balance and prevent excessive immune responses by increasing the expression of inhibitory receptor NKG2A [24]. In contrast, senescent NK cells exhibit increased secretion of pro-inflammatory cytokines, such as TNF-α [28]. With advancing age, there is a significant reduction in the release of perforin by NK cells and their ability to bind to target cells at the immunological synapse. This is associated with a decline in the capacity to release inositol trisphosphate (IP3) following interactions with target cells, as well as a delay in the hydrolysis of phosphatidylinositol 4,5-bisphosphate (PIP2), which consequently leads to a decrease in the cytotoxic activity of NK cells [29].

In conclusion, NK cells undergo significant functional and phenotypic alterations during immunosenescence, and this subpopulation imbalance and functional dissociation not only weakens immune surveillance against tumors and pathogens, but also directly participates in the development of hematological disorders (e.g., acute myeloid leukemia and myelodysplastic syndromes) through the promotion of chronic inflammation and dysregulation of the bone marrow microenvironment, making them a key immunological hub linking senescence to hematological pathologies, which is an important target for the development of immunological intervention strategies for hematological diseases. Key immunological hubs provide important targets for the development of immune intervention strategies against hematologic diseases in the elderly.

**Figure 6.**
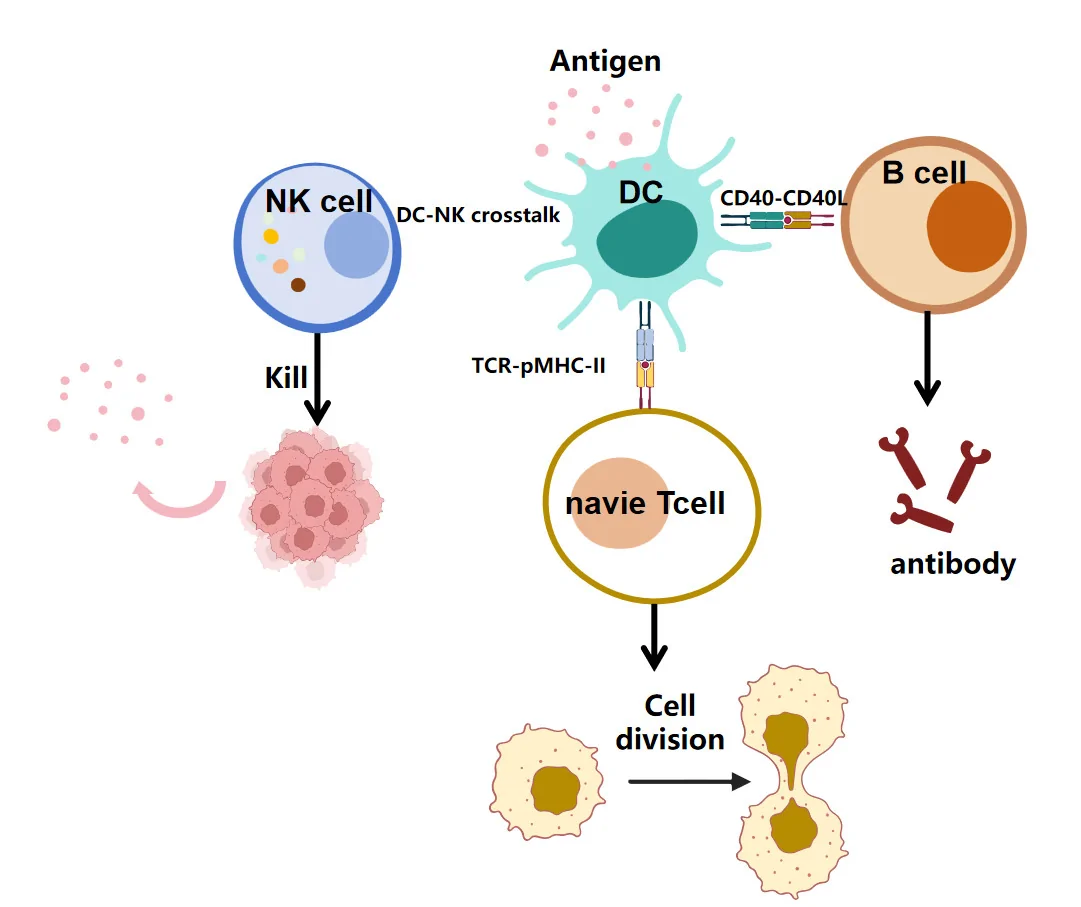
**The mechanism of action of DCs: DCs take up antigens and delivers them to T cells in the form of pMHC to activate T cells and promote their proliferation**. After coming into contact with NK cells, it enhances the ability of NK cells to kill tumor cells. It also comes into contact with B cells in the form of CD40-CD40L and promotes their secretion of antibodies.

### 3.2 Dendritic Cells (DCs): Sentinel and coordinator of the immune system

DCs are key cellular types in the immune system, primarily responsible for antigen capture, processing, and presentation, thereby activating T cell-mediated immune responses. Among the most important antigen-presenting cells (APCs), DCs are divided into myeloid DCs (mDCs) or plasmacytoid DCs (pDCs), with distinct functional activities: mDCs produce IL-12 and induce helper T cell type 1 (Th1) and cytotoxic T lymphocyte (CTL) responses, while pDCs produce IFN-α/β in response to bacteria and viruses [30]. DCs are primarily responsible for the uptake and processing of tumor antigens, presenting them to T cells in the form of antigen peptide-major histocompatibility complex class II (pMHC) complexes, thereby activating the initial T cell activation and proliferation to eliminate tumor cells. Additionally, DCs can induce anti-tumor immune pathways through the induction of antibodies or NK cells [31] (Fig. 6).

During immunosenescence, DCs undergo significant functional degradation, as evidenced by a decrease in numbers (especially mDCs subpopulations with cross-presenting capacity), dysfunction of pDCs, as evidenced by a significant decrease in the secretory capacity of type I interferon (IFN-α/β), although the number of CD123^+^ pDCs may be maintained or increased slightly and impaired antiviral response. In addition, decreased antigen uptake and processing, reduced expression of MHC class II molecules and co-stimulatory molecules result in a significant reduction in their ability to activate initial T cells [31]. The low-grade inflammatory state that accompanies aging and oxidative stress, a feature of aging, persistently activate signaling pathways such as NF-κB and MAPK; also mitochondrial function declines with aging, manifested by reduced efficiency of oxidative phosphorylation and imbalance of competence metabolism [32], which affects the cytokine secretion pattern of the DCs; in addition, the changes in DNA methylation and histone modification that come with aging [33], alterations in cytokines in the aging microenvironment [34] and enhanced recognition of self-antigens by DCs caused by elevated autoimmune risk as a result of aging, all of which lead to a tendency for DCs to produce more pro-inflammatory cytokines (e.g., IL-6, TNF-α) (Fig. 7). However, senescent DCs have impaired migratory function (e.g., abnormalities in the CCR7 signaling pathway) and reduced homing efficiency to lymph nodes, creating a paradoxical state of pro-inflammatory but low immune response [35]. These changes not only weaken the immune surveillance function against pathogens and tumors, but also exacerbate hematological disorders (e.g., acute myeloid leukemia and myeloid fibrosis) progression, becoming an important link between aging and immunodeficiency.

**Figure 7.**
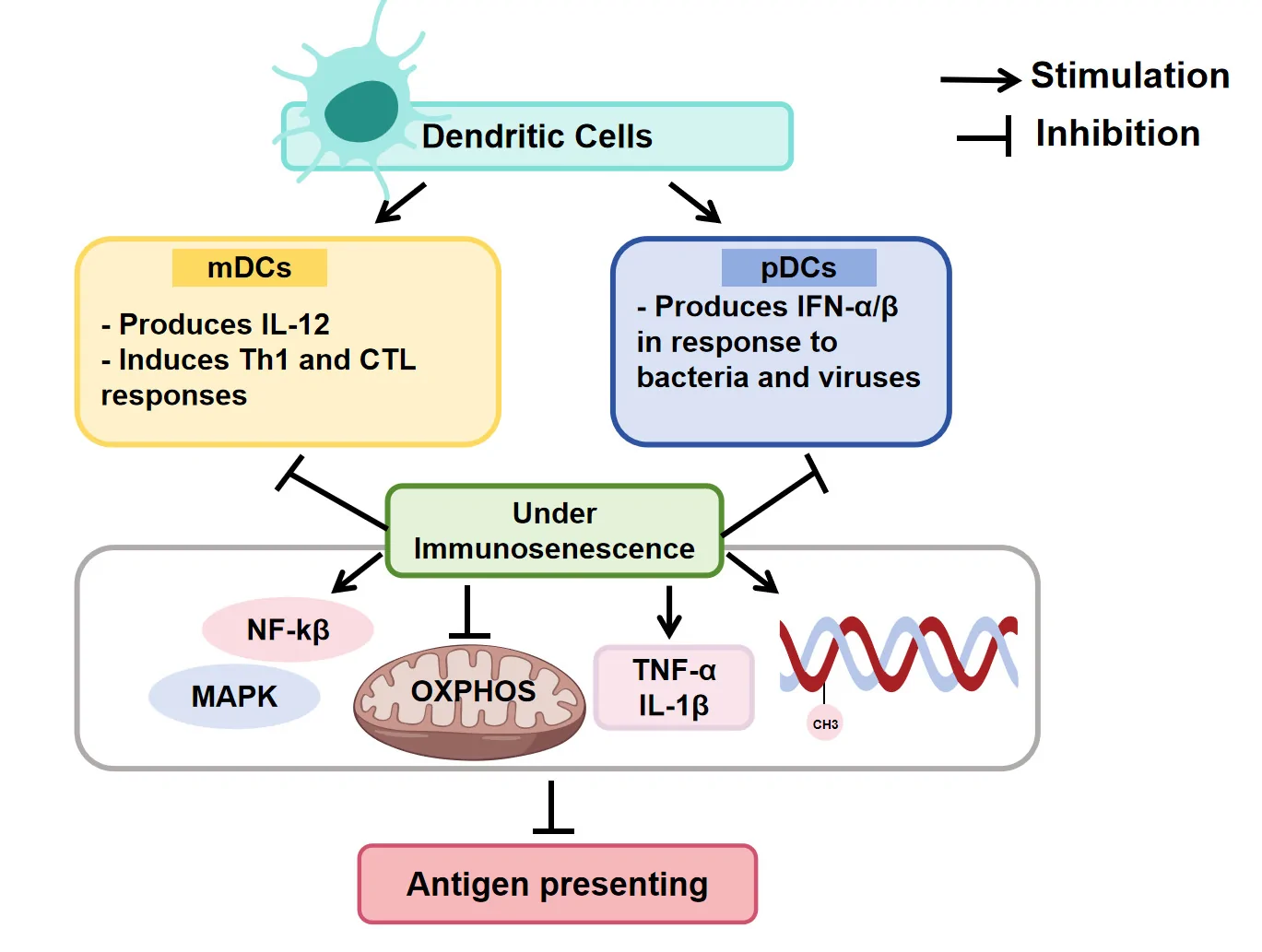
**Classification of DCs and the influence of immunesenescence on it: DCs are categorized as mDCs and pDCs, each of which has its own function**. The effect of the immunosenescence process on both ultimately results in the suppression of the ability to present antigens of DCs.

### 3.3 Neutrophils: Pioneer and scavenger of the immune system

Neutrophils are primarily composed of the myeloperoxidase (MPO) bactericidal system, producing hydrogen peroxide, halides, and MPO. Under the action of complement fragments or antibody-mediated opsonization, neutrophils play a powerful role in phagocytosing pathogens and exerting cytotoxic effects [36]. The two main phagocytic receptors expressed on neutrophils are Fc receptors (FcRs) and complement receptors (CRs). These receptors specifically bind to the Fc region of IgG antibodies or complement fragments, which mark invading pathogens to initiate phagocytosis [37]. During immunosenescence, neutrophils undergo significant functional and phenotypic changes, mainly in the form of an increase in the proportion of terminally differentiated subpopulations (e.g., CD57^+^) accompanied by an increase in functional heterogeneity but an overall decrease in their antimicrobial capacity. The specific manifestations include reduced MPO system activity, which decreases hydrogen peroxide (H_2_O_2_) and hypochlorite (HOCl) production, ultimately weakening pathogen oxidative killing capacity [36] (Fig. 8). Complement receptors (e.g., CR1) and chemokine receptors (e.g., CXCR2) become down-regulated, reducing neutrophil recruitment efficiency to inflammation sites [38]. Senescent neutrophils increasingly release pro-inflammatory factors like IL-6 and TNF-α. These cells also generate large neutrophil-derived vesicles (LAND-Vs) that inhibit complement activation through surface CD55, promoting sustained inflammation [39]. All these changes establish a dynamically regulated but overall chronic inflammatory state. Additionally, elevated expression of inhibitory molecules like PD-L1 on these cells may suppress T cell function and contribute to tumor immune escape [40]. Together, these changes lead to diminished pathogen clearance, increased risk of tissue injury, and are closely associated with the progression of hematologic diseases (e.g., AML, myelofibrosis) in the elderly.

**Figure 8.**
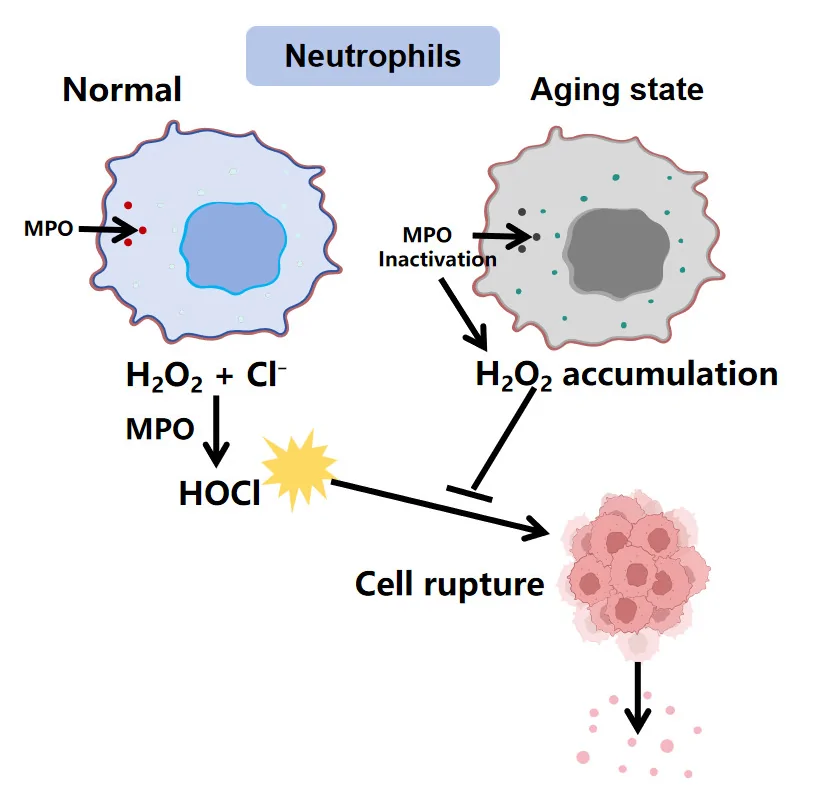
Aging leads to a decrease in the activity of the MPO system in neutrophils, thereby weakening the oxidative killing ability of neutrophils against pathogens.

### 3.4 Macrophages:Generalist of immune system

Macrophages are white blood cells found in tissues, derived from monocytes, which in turn originate from precursor cells in the bone marrow. The immune functions of macrophages mainly include two aspects: firstly, macrophages can phagocytose and kill intracellular parasites, bacteria, tumor cells, and self-aging and dead cells, thereby playing the immune defense, immune homeostasis, and immune surveillance functions of the body. Secondly, macrophages can produce pro-inflammatory cytokines, such as TNF-α, IL-1, IL-6, and IL-8. These cytokines can process and transfer antigens to T cells [29, 41, 42].

During the immunesenescence, the number of macrophages does not significantly decrease, but there are many functional changes [28]. Macrophages may exhibit different polarities according to local environmental signals, such as M1 type (pro-inflammatory) and M2 type (anti-inflammatory) [43]. Immunosenescence may affect this balance, leading to changes in the proportion of pro-inflammatory or anti-inflammatory macrophages. In studies on the number of macrophages in the spleen, lymph nodes, and bone marrow of elderly mice, we found that the aging process and the immunosuppressive microenvironment produced by aging cause macrophages to polarize towards the M2 phenotype. Since M1 macrophages are usually associated with inflammatory responses, the state of low-grade inflammation and the increase in cytokines TNF-α and IL-6 will promote the polarization of macrophages towards the M1 type. The disruption of the balance between M1 and M2 macrophages may affect their phagocytic ability and pathogen clearance efficiency, increasing the risk of infection. Additionally, aging also leads to a decline in the antigen-presenting ability of macrophages (e.g., MHC II and CD80/86), as well as their phagocytosis and digestion functions [44, 45]. It was found that after stimulation with IFN-γ, the expression of MHC II on macrophages from elderly mice is 50% lower than that on macrophages from young mice [46]. In addition, senescent macrophages carry molecules such as miR-378a through extracellular vesicles (EVs) to propagate senescence signals to other tissues, driving systemic senescence and age-related diseases (e.g., osteoporosis, metabolic disorders) [47]. In the TME, senescent macrophages tend to be M2-like polarized (TAMs), which inhibit T cell function and promote immune escape and malignant progression through mechanisms such as PD-L1 upregulation [46, 48]. Together, these changes lead to defective immune surveillance and imbalanced tissue homeostasis, which are key drivers of aging-related diseases.

**Table 2:** Effects of aging on T cells.

Cytokines	Change
**T cell subsets**	
**Navie T cells**	decreased
**Memory T cells**	increased
**Effector T cells**	decreased
**Regulatory T cells (Treg)**	increased
**Function**	
**Proliferation**	decreased
**Cytotoxicity**	decreased
**Helper function**	decreased
**Response to antigens**	decreased
**Cytokine and chemokine production**	decreased
**Surface markers**	
**CD28 expression**	decreased
**CD45RA expression**	decreased
**CD45RO expression**	increased
**CD127 expression**	decreased
**Functional markers**	
**IFN-γ**	decreased
**IL-2**	decreased
**TNF-α**	increased

## 4. Immunosenescence of adaptive immune response cells

### 4.1 T Cells: The Elite Force and Strategists of the Immune System

The development, differentiation, and maturation of T cells are inseparable from the thymus, which gradually degenerates during the aging process, leading to a decrease in the production of T lymphocytes and a decline in immune function (Table 2). The degeneration of thymus limits the output of immature T cells [49], and the gradual decrease in T cell release from the thymus leads to a reduction in the T cell pool in peripheral lymphoid organs. Meanwhile, a large number of memory T cells or long-lived naive T cells are detected in the elderly. This suggests that naïve T cells tend to differentiate to memory T cells. Moreover, the number of naive CD4+ and CD8+ T cells (CD45RA+) gradually decreases, while the number of memory phenotypes (CD45RO+) gradually increases during aging [50, 51] (Fig. 9A). Senescence of CD8^+^ T cells is particularly prominent, as evidenced by oligoclonal expansion to chronic antigens such as CMV and mitochondrial dysfunction [52].

**Figure 9.**
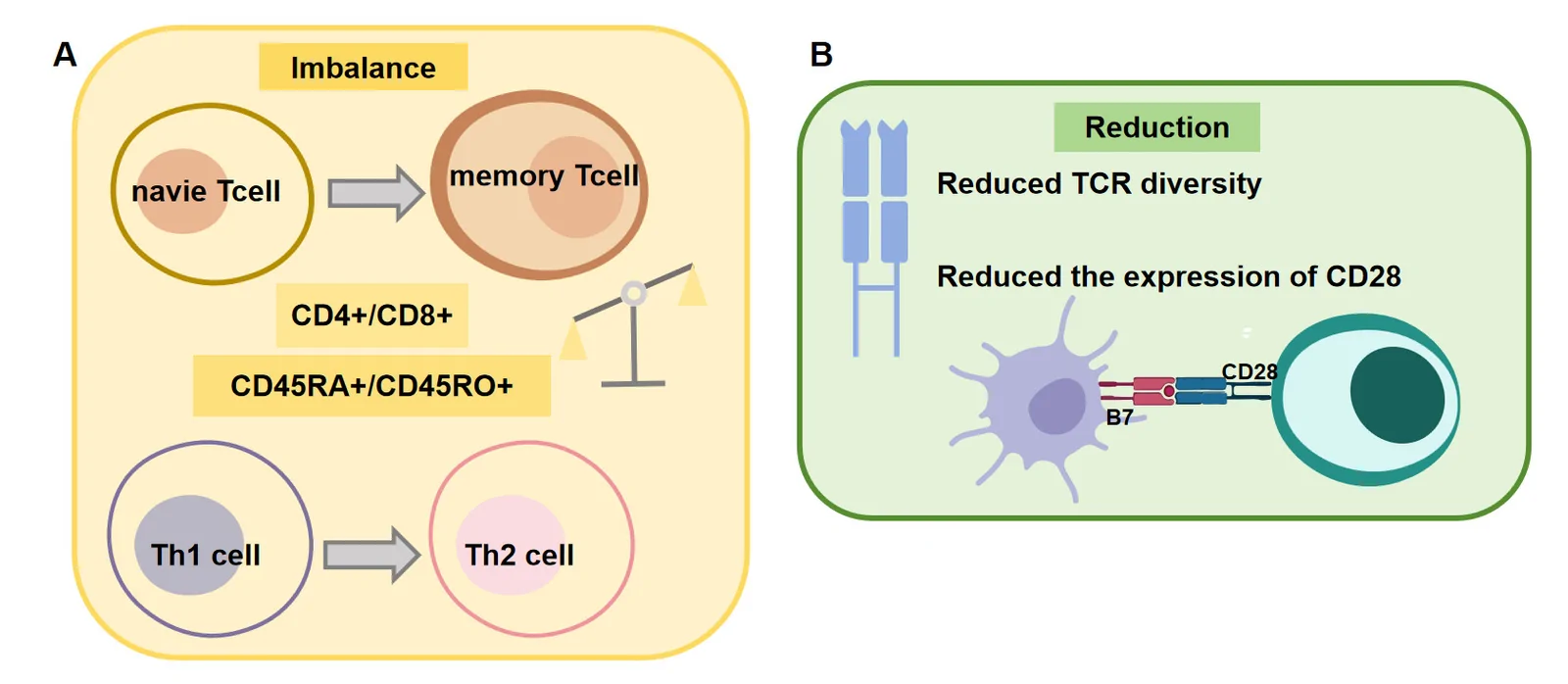
**Effects of immunosenescence on T cells**. (**A**) Navie-memory imbalance-immunosenescence promotes the expression of memory T cells and causes the imbalance of CD4+ T cell/CD8+ T cell ratio, and the CD45RA+ phenotype decreases while the memory phenotype CD45RO+ increases. Th1-Th2 imbalance- immunosenescence promotes the transfer of Th1 cells to Th2 cells. (**B**) Senescent T cells have decreased TCR diversity and decreased CD28 expression.

Additionally, one of the more important phenotypic changes in T cells in elderly individuals is related to the decreased expression of CD28, which is a sign of age-related decline in T cell function [53] (Fig. 9B). This is an anti-inflammatory protein and co-stimulatory protein expressed on the surface of T cells and is crucial for the development of T cell-mediated immune responses. Activated CD28 interacts with a protein called B7 on APCs, enabling them to co-stimulate T cells, thereby increasing the survival, proliferation, and cytokine production of T cells [54].

Chronic inflammation stimulates the secretion of pro-inflammatory cytokines such as TNF-α, IL-1β, IL-6 and IL-2 decreases. Although the decrease in T cell activation and proliferation ability leads to a decrease in IFN-γ levels [55], it does not significantly decrease due to the presence of chronic inflammation. In addition, changes in cytotoxic molecules, such as the increased secretion of perforin and granzymes, are closely related to the aging process.

Moreover, the clonal diversity of the T cell receptor (TCR) may decrease with age (Fig. 9B), limiting the T cells response to new antigens [56, 57]. The function of regulatory T cells (Tregs) may be impaired, affecting their ability to suppress excessive immune responses. Garg *et al*. confirmed the increase in Treg cell function in elderly mice, and a large number of Treg cells have been found in both elderly mice and humans. This partly explains the higher risk of tumor occurrence in elderly individuals because cancer cells can use the suppressive environment to evade the immune system [58].

With immunosenescence accompanied by low-grade inflammation, the balance of Th cells (such as Th1, Th2, Th17) may change, affecting inflammatory responses and immune protection. Among them, the imbalance of Th1/Th2 ratio shifts towards Th2 [59] (Fig. 9A). Th2 cells secrete cytokines such as IL-4, IL-5, IL-6, and IL-10, which not only promote the proliferation of Th2 cells, assist in B cell activation, participate in immune responses, but also participate in the process of inhibiting Th1 cell proliferation, a key step in immune responses. Th1 cells release cytokines such as IL-2, IL-17, IFN-γ, and GM-CSF [60], which not only mediate cellular immunity and delayed-type hypersensitivity but also play a key role in the occurrence and development of autoimmune diseases. Cytotoxic T cells may exhibit clonal expansion due to continuous antigen stimulation but may also decline in function due to cellular aging. For memory T cells, the proportion of CD4+ and CD8+ T cells shows absolute growth [61].

### 4.2 B Cells: Specialized weapons and memory banks of immune system

B cells originate from HSCs in the bone marrow (BM) [62] and mature in the spleen. B cells are responsible for secreting antibodies and play an important role in humoral immunity. B lymphocytes mainly mediate the body's specific humoral immunity, capable of recognizing thymus-dependent antigens (TD-Ag) and thymus-independent antigens (TI-Ag), completing the immune response to all antigens.

The function of HSCs declines with age, the precursor B cell pool decreases, and the development of potential differentiation B cells is damaged [62, 63]. The aging microenvironment affects the activation and maturation of B cells. In aging-accelerated mice, it has been observed that the production of TGF-β in bone marrow stromal cells decreases. Co-culturing stromal cells from young mice with precursor B cells from elderly mice leads to a decrease in the number of mature B cells [63]. These results indicate that compared with young mice, elderly mice have a reduced number of B cell precursors, pre-B cells, and immature B cell subsets in the bone marrow, and the mechanism involves a combination of cellular intrinsic changes and microenvironmental changes [62]. With increasing age, the bone marrow microenvironment undergoes various changes, including a decrease in the level of the cytokine IL-7 produced by stromal cells. IL-7 plays a key role in the survival and proliferation of B cell precursors [64, 65]. At the molecular level, aging is also accompanied by a decrease in the expression of transcription factors E2A, alternative light chains of BCR, and PAX5 [66, 67]. In the bone marrow of elderly mice, the frequency of pro-inflammatory B cell subsets-age-related B cells (ABC) increases, and these cells secrete a large amount of TNF-α, which has been proven to damage the generation of young pre-B cells [68]. Senescent B cells exacerbate “inflammatory senescence” through pro-inflammatory cytokine secretion and interfere with T cell function through the upregulation of inhibitory receptors (e.g., PD-L1, CD22), creating a vicious cycle. Notably, the accumulation of senescence-associated B cell subsets further exacerbates autoimmune tendencies and immune homeostasis.

Furthermore, aging also reduces the expression of molecules involved in immunoglobulin (Ig) class switch recombination (CSR) and somatic hypermutation (SHM), both of which are key to the production of high-affinity protective antibodies and the formation of germinal centers (GC). In elderly mice, due to the decreased expression of E47 and activation-induced cytidine deaminase (AID), B cells in the spleen have defects in isotype switching and class switching after stimulation with a mitogen, and cannot effectively produce a variety of antibody types [69]. AID, as a key enzyme that triggers DNA cleavage during the CSR and SHM processes of Ig genes [70], is crucial for the development of high-affinity antibodies and the strengthening of immune responses. Senescent B cells exhibit reduced BCR diversity and diminished SHM, resulting in a narrowed antigen recognition repertoire. Concurrently, downregulation of co-stimulatory molecules (e.g., CD27, CD40) and impaired CSR efficiency collectively compromise the generation of high-affinity antibodies. Together, these changes manifest as poor vaccine response (e.g., >50% reduction in antibody potency to influenza vaccines), increased auto-antibodies, and promotion of age-related diseases (e.g., chronic inflammation, hematologic malignancies).

## 5. Immunosenescence and Hematological Diseases

Hematological diseases can be classified based on various criteria, one of the main ways is to distinguish between neoplastic hematological diseases and non-neoplastic hematological diseases according to whether they involve tumorous (uncontrolled proliferation of abnormal cells). Neoplastic hematological diseases refer to diseases that involve the uncontrolled proliferation of abnormal blood cells, usually divided into the following categories: leukemia, lymphoma, multiple myeloma, and myelodysplastic syndromes [70]; non-neoplastic hematological diseases refer to diseases that do not involve abnormal cell proliferation, which may be caused by genetics, autoimmune reactions, infections, malnutrition, or other factors, including: anemia, coagulation disorders, myelodysplastic syndromes (MDS), and autoimmune hematological diseases [71].

Among the many factors that influence the development of hematologic diseases, aging is one of the key players. Functional remodeling of immune cells during immunosenescence (e.g., reduction of T cell pool diversity, dysregulation of B-cell antibody response, pro-inflammatory polarization of myeloid cells, and decline in NK cell toxicity) not only leads to a general decline in the body's defense capacity, but also directly participates in the initiation and evolution of hematologic diseases by creating a chronic inflammatory microenvironment, weakening immune surveillance, and facilitating the escape of malignant clones. Particularly noteworthy is the aberrant interactions between senescent immune cells and HSC and stromal cells, which form a vicious circle: senescent immune cells drive inflammation and fibrosis in the bone marrow microenvironment through the secretion of SASP factors (e.g., IL-6, TNF-α), accelerating the decline of the HSC function; the skewed differentiation of lymphoid lineage associated with HSC senescence further exacerbates immune cell imbalance. This bidirectional regulatory network provides new perspectives for understanding the high prevalence and treatment resistance of hematologic diseases in the elderly. Thus, immunosenescence is a key player in the development of hematologic diseases. Based on this, the following section focuses on several typical hematologic diseases and systematically analyzes how immuno-senescence affects their onset, progression, and therapeutic response through specific molecular pathways.

## 6. Effect of immunosenescence on non-neoplastic blood diseases

### 6.1 Aging in Autoimmune Hemolytic Anemia (AIHA)

AIHA is an anemia caused by the immune system abnormally, leading to auto-antibodies attacking and destroying red blood cells. Its characteristics include hemolysis, that is, the breakdown of red blood cells (RBCs), which occurs together with auto-antibodies and/or complement, as well as activated macrophages, T lymphocytes, and cytokines that contribute to the process. All these immune parameters change with age, and immunosenescence is one of the pathological mechanisms related to autoimmunity [72, 73].

The characteristic of AIHA is the production of auto-antibodies, and B cells play a key role in the production of antibodies. The proportion of CD11c+ ABCs is elevated in senescent B cells, and these cells have a tendency to spontaneously produce auto-antibodies (e.g., anti-erythrocyte antibody IgG), which directly trigger erythrocyte destruction. Some data suggest that elderly patients are accompanied by a more active B cell/plasma cell response with a more severe hemolytic phenotype [74]; most auto-antibodies are directed against senescent erythrocyte membrane proteins (Band 3), and with accelerated erythrocyte senescence, the B cells continue to be activated by the relevant antigens, resulting in aggravation of hemolysis [75]; although the AID enzyme activity of senescent B cells is reduced [69], the auto-antibodies (e.g., anti-spectrin antibodies) can still be produced in large quantities through a non-T-cell-dependent bypass pathway, all of which leads to the abnormal expansion of autoreactive B cells. In addition, the number and receptor diversity of B cells decrease, and the amount of antibodies produced also decreases, increasing the possibility of infection [69]. The combined action of multiple factors leads to an increased incidence of AIHA with age.

Th17 cells can induce B cells to produce auto-antibodies by producing B cell chemoattractant CXCL-13, which may lead to an increase in auto-antibodies in AIHA [76, 77]. The proportion of Th cell subsets may change during the aging process. In addition to the imbalance of Th1/Th2 ratio shifting towards Th2 [59], in elderly AIHA patients, naive CD4+ T cells tend to develop into Th9 cells, and the secretion of IL-9 also increases. Th9 cells are a newly discovered subset of CD4+ T cell differentiation that participates in the body's immune response by secreting IL-9. In patients with autoimmune diseases, the proportion of Th9 cells in the blood and the content of IL-9 undergo significant changes, and Th9 cells are closely related to the occurrence and development of autoimmune diseases [78]. Additionally, some studies have shown that the content of IL-4 in the peripheral blood of elderly AIHA patients is much higher than that in the healthy control group, which may be due to the involvement of Th2 cells and IL-4 in the pathogenesis of AIHA. Studies have found that the high proportion of Th17 in the peripheral blood of AIHA patients is positively correlated with the disease activity process, and this increase in quantity also promotes the secretion of IL-6, IL-21 and other cytokines by Th17 [79]. In Treg cells, senescence induces destabilized FoxP3 expression and reduced IL-10 secretion, impairing their ability to suppress autoreactive T cells (e.g., Th17). This culminates in loss of immune tolerance to erythrocyte antigens [80]. In contrast, senescence-associated inflammatory factors (e.g., IL-6) drive macrophage differentiation toward pro-inflammatory M1 type, with up-regulated expression of FcγRⅠ (CD64) on their surface and 3-5-fold enhanced phagocytosis of IgG-coated erythrocytes. In conclusion, immunosenescence exacerbates the pathological process of AIHA, creating therapeutic challenges through a triad of mechanisms: increased autoantibody production, impaired immune tolerance, and hyperactivated phagocytic function of macrophages.

The treatment methods for AIHA mainly include corticosteroid therapy, immunosuppressive therapy, splenectomy, immunoglobulin therapy, and red blood cell transfusion [81]. Senescent B cells have downregulated CD20 expression and are apoptosis resistant (high BCL-2 expression), leading to reduced therapeutic efficiency of CD20-targeted therapies such as rituximab [82, 83]. In a multicenter retrospective study (GIMEMA group, median age 68 years), elderly AIHA patients receiving rituximab had significantly lower overall response rates (ORR 55-65%) and complete remission rates (CR 20-25%) than a younger cohort (ORR 75-85%, CR 40-50%), and had a higher rate of relapse [84]. Critically ill patients may not tolerate the delayed onset of action of rituximab (median: 15 days). Silencing of glucocorticoid receptor (GR) methylation in senescent T cells impairs hormonal suppression of autoimmune responses. In an integrative analysis of methylation profiles of aged mouse and human CD8^+^ T cells, CpG islands in the promoter region of NR3C1 (the gene encoding the GR) were found to be significantly hypermethylated in senescent/terminally differentiated T cells and negatively correlated with the level of GR mRNA and protein expression; when the methylation was reversed by 5-aza-dC dmethylating agents, GR expression was restored and T cells were again sensitive to dexamethasone-induced apoptosis [85]. Prednisone is the first-line therapeutic agent for AIHA, but elderly patients have decreased sensitivity to hormones, often requiring higher doses or longer use, which in turn exacerbates the risk of infections (e.g., pneumonia, herpes zoster), and long-term remission is not possible in about 30%-50% of elderly patients who are susceptible to relapse after hormone tapering. Complement activation is a key mechanism of hemolysis in AIHA, but aging leads to abnormal function of complement regulatory proteins (e.g., CD55), and existing complement inhibitors (e.g., eculizumab) have limited effect on warm-antibody-type AIHA and may increase the risk of meningococcal infections [86], and patients with cold-antibody-type AIHA (CAS) are more dependent on the complement pathway, but elderly patients are poorly tolerated to the treatment due to the many complications. Second-line drugs (e.g., cyclophosphamide, azathioprine) control hemolysis through extensive immunosuppression, but elderly patients are more likely to develop hematopenia (e.g., thrombocytopenia, neutrophil deficiency) due to HSC senescence and bone marrow microenvironmental fibrosis). Long-term treatment may induce secondary myelodysplastic syndromes (MDS) or infections [87, 88]. Overall, AIAH is characterized by high individual heterogeneity, lack of stratified guidance on the characteristics of elderly patients in the current guidelines, and multiple dilemmas in the use of conventional therapy in elderly patients in the setting of immunosenescence. Combination therapies (e.g., rituximab and bortezomib) may improve remission rates, but the differences in drug metabolism and the risk of complications in elderly patients still need to be further evaluated. Future directions need to strike a balance between efficacy and safety, targeting senescent immune cells and modulating the bone marrow microenvironment.

### 6.2 Aging in Myelodysplastic Syndromes (MDS)

MDS is a HSC disease defined by dysplasia of bone marrow cells and ineffective hematopoietic production [89], characterized by clonal dominance of HSC, reduced blood cells, dysplasia of BM cells, and ineffective hematopoiesis [90, 91]. MDS is caused by defective HSC reproduction and is defined by somatic mutations and chromosomal abnormalities that affect several key cellular functions, including epigenetic plasticity, DNA repair, cohesin complexes, ribosomes, and spliceosome functions, as well as immune signaling [92-94].

The greatest risk factor for developing MDS is advanced age. It is well known that the immune and hematopoietic systems undergo dysregulation and decreased function with aging [95]. From the perspective of the hematopoietic system, aging affects the HSC niche and the clonal selection pressure in the bone marrow microenvironment, changing the interaction between HSCs and the microenvironment, thus enabling HSC clones with specific genetic (such as mutations in DNMT3A, TET2, or ASXL1 genes, which may endow them with growth advantages or anti-apoptotic capabilities, thus obtaining clonal advantages during immunosenescence) or phenotypic characteristics (such as increased cell cycle activity or altered cell surface markers) [96-98], which may affect their localization and self-renewal capacity in the bone marrow to gain advantages, promoting the expansion of abnormal clones, suppressing normal hematopoiesis, increasing genetic instability, or promoting immune escape mechanisms, and promoting the development of MDS.

From the perspective of the immune system, the increase in myeloid-derived suppressor cells (MDSC) during aging, which secrete immunosuppressive molecules such as IL-10 and TGF-β, inhibits T cell activity, while the reduced migration capacity and antigen presentation efficiency of DCs affect T cell activation. The imbalance of T cell subsets, the increase in memory T cells, and the subsequent dysfunction of T cells lead to a decline in the ability of T cells to fight infections, insufficient defense against pathogens, and increased infection risk. And senescent T cells have a narrowed receptor pool and reduced ability to recognize and clear malignant hematopoietic stem cells, leading to early MDS clones evading immune clearance.

B cells produce dysfunctional antibodies during immunosenescence, affecting the defense against infections and the immune clearance of tumor cells. Auto-antibodies activate complement and further damage the hematopoietic microenvironment. The cytotoxicity and cytokine production of NK cells decrease with aging, reducing the clearance of abnormally proliferating MDS cells. Accumulation of a terminally differentiated subpopulation of CD57^+^ NK cells: these NK cells secrete less IFN-γ but more proinflammatory factors (e.g., TNF-α), which exacerbate inflammation but are unable to control the malignant clone. Tregs may dysfunction in MDS, failing to effectively suppress autoimmune reactions, potentially leading to immune regulation imbalance. The decline in immune surveillance also reduces the clearance of malignant clones, allowing the continuous expansion of MDS clones. The increase in pro-inflammatory cytokines may promote the progression of MDS and increase the risk of transformation into acute myeloid leukemia (AML) [99-102]. In summary, immunosenescence contributes to the onset and progression of MDS through multiple dimensions of defective immune surveillance, an inflammatory microenvironment that promotes malignant clonal proliferation, and disruption of the HSC ecosystem, leading to therapeutic resistance.

Elderly MDS patients may require more frequent red blood cell and platelet transfusions, but frequent transfusions may lead to iron overload or other complications [103], and senescence-associated HSC differentiation skewed (toward the myeloid lineage) and EPO receptor sensitivity declined, which leads to reduced efficacy of erythropoietin (EPO), and predispose to transfusion-dependent anemia [104]. Demethylating drugs (e.g., azacitidine or decitabine), as the most commonly used treatment [105], abnormal DNA methylation patterns (e.g., TET2 mutations) in senescent T cells and myeloid cells lead to reduced sensitivity to demethylating drugs (azacitidine/decitabine), with only 30-40% of patients achieving sustained remission [106, 107]. In addition, long-term Hypomethylating Agent (HMA) treatment may accelerate the expansion of TP53 mutant clones [108], especially when accompanied by depletion of immunosenescent CD8^+^ T cells and the inability of the immune system to clear drug-resistant subclones [109]. IL-6 and TNF-α secreted by senescent macrophages maintain immunosuppression through the STAT3 pathway, counteracting inefficient immune checkpoint inhibitor (ICI) effects. Clinical data show that ICI single-agent efficacy is <15% in elderly MDS patients [110], and the upregulation of PD-1/CTLA-4 expression in senescent T cells, but loss of proliferative capacity due to terminal differentiation (CD28-CD57^+^), and difficulty in restarting the immune response with ICIs (e.g., nabulizumab) have contributed to the inefficient ICI response in elderly MDS patients. In addition, conventional hematopoietic stem cell transplantation is a potential cure for MDS, but a study showed that in elderly patients, the cumulative incidence of acute Graft-versus-host disease (GVHD) grades II-IV in the ≥60-year-old group compared with the 40-59-year-old group increased from 29.2% to 33.2% due to reduced thymic output and delayed recovery from infections as a result of immunosenescence. The cumulative incidence of acute GVHD grades II-IV in the ≥60-year-old group increased from 29.2% to 33.3%, and the cumulative incidence of chronic GVHD from 16.9% to 41.0% compared with that in the 40-59-year-old group [111]. In conclusion, immunosenescence significantly undermines the efficacy of current MDS therapeutic strategies through epigenetic disorders, inflammatory microenvironment, and dysregulation of hematopoietic-immune interactions. Breakthroughs need to be focused on the precise regulation of senescent immune cells rather than the suppression of malignant clones alone.

### 6.3 Aging in Primary Immune Thrombocytopenia (ITP)

ITP is defined as isolated thrombocytopenia (peripheral blood platelet count <100×10^9/L) in the absence of a confirmed cause, and is still an exclusion diagnosis [112, 113]. This is an autoimmune disease characterized by a significant decrease in platelet count, leading to bleeding tendencies, such as petechiae (small bleeding points), bruises, epistaxis, or gum bleeding. Over the past few decades, the incidence of ITP in the elderly has gradually increased, with the incidence in people over 60 being twice that of those under 60 [114].

The pathophysiology of ITP is complex and still not fully understood. The traditional concept is that antibody-coated platelets are prematurely destroyed in the spleen, liver, or both through interactions with Fcγ receptors [113, 115-117]. Auto-antibodies can also induce complement-mediated or delipidation-induced platelet destruction and inhibit megakaryocyte function [118, 119].

During immunosenescence, the ability of B cells to produce antiplatelet auto-antibodies is enhanced, and although it has also been stated that antiplatelet antibodies are not detected in up to 50% of patients, macrophages in their spleens and livers may be more active in removing platelets labeled by auto-antibodies, exacerbating thrombocytopenia [120]. In elderly ITP patients, not only does platelet production decrease, but platelet antibodies produced by B cells exacerbate platelet destruction, leading to a significant reduction in platelets from both the source and destination directions. The propensity for macrophage M1-polarization enhances phagocytosis of IgG-coated platelets, thereby exacerbating platelet hyperactivation. In vitro experiments showed that the phagocytosis efficiency of CD64 high-expressing senescent macrophages on IgG-encapsulated platelets was increased by about 1.8-fold compared with that of young controls [120, 121]. Reactive oxygen species (ROS) released from senescent platelets further activate macrophages [122, 123] (Fig. 10), forming a vicious cycle of “inflammation-phagocytosis”. Secondly, the decrease in T cells and the imbalance of Th cell subsets, including the shift of Th cells towardsTh1 and Th17 phenotypes, as well as the reduction in the number and function of Treg cells, may drive the autoimmune process [119, 124, 125]. The decrease in Tregs weakens the inhibition of autoreactive B cells, which may exacerbate the course of ITP [126, 127]. That is, an imbalance in the Treg/Th17 ratio upsets the balance of immune tolerance and promotes autoreactive T cells to attack platelets and megakaryocytes. DCs may have reduced antigen presentation ability during immunosenescence, and DCs are the most important APCs in the body, playing a key role in the pathogenesis of ITP. DCs from ITP patients stimulated with anti-CD70 antibodies can lead to an increase in conventional CD4+CD25- T cells while reducing the production of Tregs [128]. NK cells were similarly confronted with a failure of immune surveillance, and senescent NK also weakened negative regulation of Th17 cells, indirectly promoting an autoimmune response. In conclusion, immunosenescence drives the progression of ITP through a combination of T/B cell dysfunction, macrophage hyperphagy, and defective NK cell surveillance, and leads to treatment resistance.

**Figure 10.**
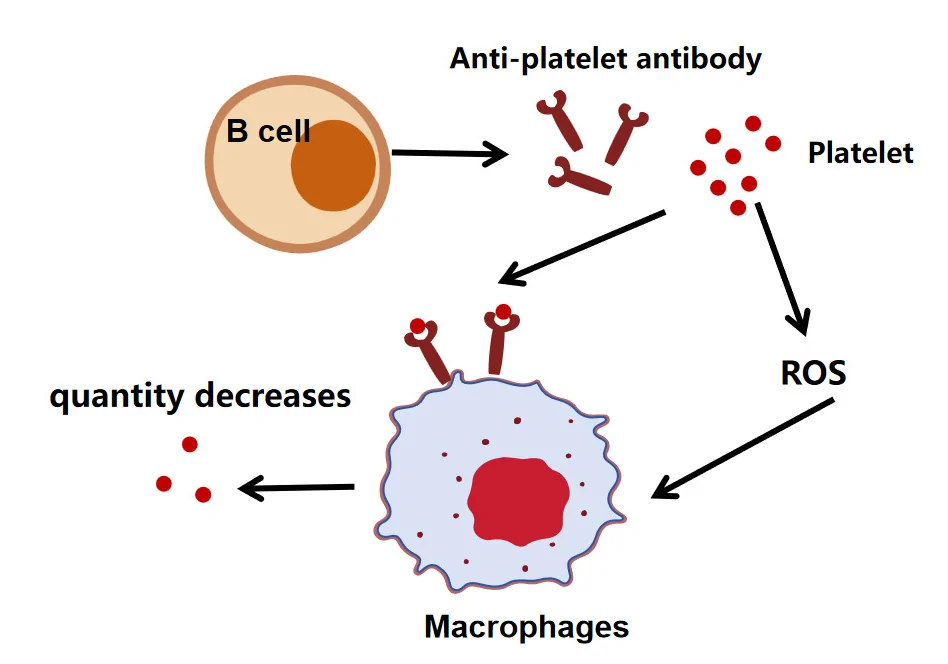
**Platelet antibodies produced by senescent B cells bind to platelets**. Macrophages clear this complex, leading to thrombocytopenia. Moreover, senescent platelets also release ROS to enhance the function of macrophages.

Elderly patients with ITP have a reduced initial response rate to glucocorticoid therapy of 50-60% and a relapse rate of >70% within 6-12 months due to decreased thymic output and CD8^+^T cell depletion. Long-term glucocorticoid use in patients aged ≥65 years triggers exponential rises in diabetes, osteoporosis, and neuropsychiatric complications, often mandating premature therapy discontinuation with consequent efficacy limitations. With rituximab, faced with a situation similar to AIHA, the rate of sustained remission in elderly patients is only 20-30%, which is significantly lower than that in the younger population (≈50%). Secondly, elderly patients have fibrosis of the bone marrow microenvironment, senescence of hematopoietic stem cells, and decreased sensitivity of megakaryocytes to TPO signaling; real-world data show that less than 35% of the >65-year-old group had sustained platelets of ≥50×10^9/L at 6 months, and they often need to be maintained with a combination of other medications [129]. And TPO-RA is associated with excessive arterial thrombosis, an important consideration in older patients with underlying atrial fibrillation and peripheral arterial disease. The bleeding risk in elderly ITP patients increases significantly with age [130], and studies have shown that the annual bleeding risk in patients over 60 can reach 13%, while in patients under 40, it is only 0.4% [131]. In addition, warm-antibody ITP (the majority) responds poorly to complement inhibitors (e.g., eculizumab) and older patients are at increased risk for infections (e.g., meningococcal). Immunosenescence operates through a triad of mechanisms—functional exhaustion of immune cells, diminished hematopoietic reserve, and heightened toxic susceptibility, which collectively impose significant limitations on current ITP treatment strategies in elderly patients, characterized by low response rates, high relapse incidence, and elevated toxicity. It is necessary to focus on the precise intervention of senescent immune cells (e.g., targeting CD38^+^ plasma cells or PD-1^+^ T cells) and to conduct individualized clinical trials for elderly patients.

## 7. Effect of immunosenescence on neoplastic blood diseases

### 7.1 Aging in Acute Myeloid Leukemia (AML)

AML is a clonal disease originating from a rare group of leukemia stem cells [132], and it is a heterogeneous hematological malignancy [133]. In recent years, significant progress has been made in understanding the biology of AML, which has greatly affected the diagnosis and prognosis of AML, leading to a new risk stratification of AML [134]. The pathogenesis of AML is mainly related to the abnormal clonal proliferation of hematopoietic progenitor/stem cells [135, 136]. The incidence of AML increases with age, and most AML patients are over 65 years old.

Immunosenescence provides the ground for the development, progression and drug resistance of AML through the triple mechanism of “cellular subpopulation remodeling-functional depletion-immunosurveillance deficiency”. As mentioned earlier, during immunosenescence, NK cell subsets are remodeled and CD56dim terminally differentiated subsets accumulate, leading to a decrease in overall killing capacity, downregulation of activated receptor expression, and a reduction in the number of key effector molecules, which are unable to effectively clear AML progenitor cells. Clinical studies have shown that NK cell defects in elderly AML patients are significantly associated with disease progression and shorter overall survival [137]. CD8+ T cells, with high expression of PD-1, TIM-3, LAG-3, and CD57, have an elevated proportion of CCR7-CD45RA+ terminal effector cells, fewer naïve cells, and decreased TCR diversity and tissue-resident memory T cells (TRM). Although cytokines can still be secreted, sustained antigenic stimulation leads to depletion and immune surveillance failure, providing a window for AML clonal escape [138]. Macrophage pro-inflammatory polarization, the formation of SASP phenotype, enhance the immnosuppressive microenvironment, and its secretion of IL-6, TGF-β and other factors, which can directly promote the survival and drug resistance of leukemia stem cells (LSC), and studies have shown that AML patients with elevated levels of IL-6 in the bone marrow is significantly correlated with the risk of chemotherapy resistance and relapse, and that patients with high levels of IL-6 have shorter progression-free survival (PFS) [139]. Decreased numbers of DCs and decreased capacity for antigen uptake and cross-presentation lead to insufficient initial CD8+ T cell activation. The number and inhibitory function of MDSC (especially polymorphonuclear MDSC, PMN-MDSC) and Treg in the bone marrow of the elderly are enhanced, and they inhibit the activity of T cells and NK cells through the mechanisms of arginase-1 (Arg-1), indoleamine 2,3-dioxygenase (IDO), and IL-10, and synergize with the establishment of the immune escape microenvironment of AML, and the high MDSC/Treg ratio of AML patients is not favorable to the chemotherapy. AML patients with high MDSC/Treg ratios have significantly lower response rates to chemotherapy and immune checkpoint inhibitors (e.g., PD-1 antibodies) and an elevated risk of relapse [140].

Analyzed in terms of treatment strategies, demethylating drugs, as conventional epigenetically-targeted therapies, usually need to be used for at least 6 cycles to show efficacy, but many elderly patients experience non-response or relapse after treatment. A study analyzing 2,263 elderly patients with primary AML treated with HMA between 2005-2015 in the United States found that only 42% of the patients were able to receive more than 4 cycles of treatment [141]. In addition, conventional chemotherapy has not been successful in elderly AML patients, who can only tolerate reduced-dose regimens due to HSC aging, bone marrow microenvironmental fibrosis, and complications; and even then, CR rates of 60-80%, 5-year overall survival rates of <10%, and extremely high relapse rates are observed. The cumulative incidence of acute GVHD after transplantation is 33% and chronic GVHD 41%, with a significantly higher non-relapse mortality rate due to reduced thymic output and delayed T/NK cell reconstitution, and only a very small percentage of patients >65-70 years of age complete transplantation [142]. For immunotherapy, CD8^+^ T cells enter a state of senescence/exhaustion (IES phenotype), with a response rate to PD-1/PD-L1 monoclonal antibody monotherapy of <15%; the inflammation-exhaustion circuit caused by immunosenescence cannot be reversed by blocking PD-1 alone [143], and NK cell relay therapy also results in autologous or allogeneic GVHD due to the downregulation of the activation receptor for NK cells and decreased cellular toxicity in immunosenescence [137]. NK cell relay therapy is also limited by the downregulation of NK cell activation receptors and decreased cellular toxicity during immunosenescence, resulting in limited efficacy of autologous or allogeneic NK infusion [137]. CAR-T therapy, as a popular novel therapeutic tool for AML, has been shown to be associated with the formation of an immunosuppressive niche in the enriched senescent macrophages, MDSC, and Tregs, which hinders the infiltration of CAR-T or TCR-T cells. The low volume of T cell acquisition and the limitation of T cell expansion have made it difficult to land the solid CAR-T therapy in AML [143]. In summary, immunosenescence creates a therapeutic trifecta of bottlenecks—diminished hematopoietic reserve, immune cell exhaustion, and immunosuppressive microenvironments, which collectively render current AML therapies in elderly patients markedly limited by shallow remissions, severe toxicities, and frequent relapses. This necessitates prioritized targeting of senescence-associated pathways (e.g., CD84, MLR). The breakthrough requires precise intervention focusing on aging-related pathways (e.g., CD84, Menin) and the development of dynamic monitoring technology to realize individualized treatment.

### 7.2 Aging in Multiple Myeloma (MM)

MM is a blood cancer that affects plasma cells. Plasma cells are a type of B lymphocyte responsible for producing antibodies. In multiple myeloma, abnormal plasma cells (known as myeloma cells) proliferate clonally in the bone marrow [144-147].

In certain cases, B cells can produce antibodies against myeloma cells, which can neutralize tumors or enhance the attack of NK cells and macrophages on tumor cells through antibody-dependent cellular cytotoxicity (ADCC) [148]. While senescent B cells have decreased antibody affinity, immune paralysis and hypo-gammaglobulinemia increase the risk of infection; senescent plasma cells themselves can secrete SASP (IL-6, IL-8, TGF-β), forming a tumor-promoting microenvironment [149]. NK cell dysfunction leads to the inability to clear early abnormal plasma cells and promotes the transformation of MGUS to MM. The antigen presentation function of DCs is dysfunctional, weakening their ability to activate T cells, and the number and function of T cells are abnormal, with CTLs having decreased ability to recognize tumor-specific antigens on the surface of MM cells, reducing their killing power on tumor cells [150]. BM-derived dendritic cells (BM-DCs) from multiple myeloma patients directly interact with tumor cells, downregulate proteasome subunits, and endow resistance to CD8+ T cells [151], thus accelerating immune escape. Data have shown that elderly patients with T cell depletion at baseline have an objective remission rate of <15% for immunotherapies such as T cell crossover (TCE) and CAR-T [152].

MM tumor cells re-shape the tumor microenvironment, establishing an immunosuppressive tumor microenvironment, which promotes the occurrence and development of MM, and immunosenescence accelerates this process [153, 154]. With immunosenescence, the number or function of Tregs increases or becomes dysfunctional, leading to excessive immune suppression and reducing the immune response against MM [155]. Tumor-associated macrophages (TAM) polarize toward M2, secrete IL-10 and TGF-β, and maintain the immunosuppressive microenvironment. Senescent bone marrow stromal cells and immune cells secrete SASP factors (IL-6, IL-8, IL-10, TNF-α, TGF-β), which not only directly support MM cell survival, but also recruit and activate MDSC, TAM, and Treg, forming a positive feedback immunosuppressive loop [149]. NK cells in the TME exhibit an exhausted phenotype, characterized by the downregulation of NK cell activation receptors and the upregulation of PD-1 [156-158]. The expression of HLA Class I is reduced, but the reduced expression of activating receptors decreases the cytotoxic ability of NK cells and reduces lysis by PCs [159, 160]. The ligands expressed by PCs that help NK cell recognition are also reduced in MM, weakening the clearance of MM cells. The abnormal clonal expansion of B cells and plasma cells hinders the secretion of antibodies by normal plasma cells, accelerates the transformation of B cells into malignant plasma cells, and secretes monoclonal antibodies uncontrollably, thus promoting the development of MM [161, 162]. The continuous increase in low-level pro-inflammatory factors such as IL-1, IL-6, TNF-α, and CRP also increases the susceptibility of the elderly to MM. In summary, immunosenescence itself leads to a failure of “immune surveillance”, which is further driven by MM cells and manifests itself in the phenomenon of “immune scarring”: T cell dysfunction persists for years, even during periods of deep remission.

Elderly patients have reduced thymic output, significantly fewer initial and central memory T cells in the peripheral blood, and a low percentage of “young” T cells available for CAR-T preparation, resulting in a diminished end-product; clinical data show that the objective remission rate of CAR-T in patients >65 years old with R/R MM is approximately 20% lower and the percentage of durable remissions is lower than that in younger patients [152]. Second, the efficacy of immunomodulatory drug IMiDs is limited by T cell depletion; lenalidomide/pomalidomide is dependent on T cell activation (via CRBN-E3 ubiquitin ligase), but senescent T cells have decreased CD28 expression and shortened telomeres, leading to attenuated proliferative and cytotoxic responses, and a 30-50% reduction in the clinical remission rate [163, 164]. Bortezomib/ Carfilzomib works by inhibiting the NF-κB pathway, but elderly patients have a 2-3-fold higher incidence of thrombocytopenia and neutrophil deficiency due to HSC senescence and microenvironmental fibrosis [165, 166], which leads to increased toxicity of proteasome inhibitors (PIs). Down-regulation of CD38 expression in senescent B cells and plasma cells and shearing of CD38 extracellular segments by MDSC via ADAM17 results in target loss, leading to immune escape from monoclonal antibody analogs (CD38 monoclonal antibody dalteplumab) [167]. In addition, immunosenescence results in the inability of terminally differentiated T cells to restart, which results in ICI failure, with PD-1 antibody single-agent efficacy of <10% [168], and TCR signaling defects in senescent T cells limiting efficacy even when targeting bispecific antibodies. Stem cell transplantation for ASCT is also limited by poor tolerance in older patients and loss of microenvironment to aging, with transplantation-related mortality (TRM) elevated to 15%-20% in patients >65 years of age due to insufficient HSC reserve and delayed immune reconstitution [169], increased secretion of IL-6 and TGF-β by senescent bone marrow stromal cells (BMSCs) inhibits autologous stem cell implantation and promotes residual lesion survival. Therefore, for elderly MM patients, it is necessary to assess the physical condition through assessment systems such as the IMWG's GA assessment system and the Mayo frailty model to guide individualized treatment. Frailty assessment includes variables such as age, biomarkers, complications, disease staging, frailty status, physical capacity, etc [170-172].

## 8. SUMMARY

Within the context of immunosenescence, alterations in the immune system and the impact of therapeutic strategies on hematologic malignancies exhibit both shared and disease-specific features. The following analysis will examine these aspects through two lenses: disease-specific mechanisms in AML and MM, and common pathways potentially generalizable to other hematologic neoplasms.

Firstly, TRM reduction as well as FLT3, IDH mutations and the CD47-SIRPα axis are AML-specific therapeutic targets [173]; MM plasma cell escape, secretion of sBCMA to neutralize the effects of CAR-T as well as the association of extra-marrowed lesions and bone disease with GPRC5D, LILRB4, and SEMA4A are emerging MM-specific targets [174].

T cell functional depletion and immune checkpoint suppression, the intrinsic mechanisms of which include things like senescent T cells (especially CD8^+^) exhibiting up-regulation of PD-1, TIM-3, reduced TCR diversity, and an increased proportion of terminal differentiation (CD28-CD57^+^), leading to decreased anti-tumor activity and limited ICI due to the inability of senescent T cells to effectively restart, similar mechanisms are seen in chronic lymphocytic leukemia (CLL) and diffuse large B cell lymphoma (DLBCL), suggesting that T-cell depletion is a common obstacle to immunotherapy for hematological tumors [175]. The decline in NK cell function and the potential of CAR-NK, in B-cell malignancies (e.g., B-ALL), has shown a 73% remission rates and a better safety profile than CAR-T. MDSC and the inflammatory microenvironment [176], MDSC expansion and the pro-inflammatory microenvironment are equally critical in lymphoma and myelofibrosis [177, 178]. Senescence-associated clonal hematopoiesis (CH) drives the accumulation of TP53/DNMT3A mutations, leading to resistance to targeted therapies, and CH monitoring is applicable to all hematological oncology patients receiving long-term immunomodulators or chemo-therapy.

## 9. Conclusions and Prospects

With the continuous development of an aging population, the incidence of various hematological diseases has increased, and the role of immunosenescence in diseases is becoming more and more important. Many studies have shown that during immunosenescence, various immune cells undergo different changes, which affect the onset of diseases from a mechanistic perspective and also bring changes to treatment strategies.

The maintenance of normal hematopoiesis depends on a normal immune microenvironment. During aging, due to the imbalance of DCs and T cells, normal immune tolerance is destroyed, leading to the production of antibodies against normal blood cells, resulting in a significant increase in the incidence of autoimmune hematological diseases (ITP and AIHA) in the elderly population. Aging leads to defective HSC reproduction and affects several key cellular functions of somatic mutations and chromosomal abnormalities, thus increasing the incidence of MDS in the elderly population. In addition, the decline in immune surveillance function is one of the important reasons for the occurrence and development of tumor cells. Tumor cells themselves lower antigen expression through various ways to achieve immune escape. The deterioration of immune cell function is another important mechanism of tumor immune escape. The phenomenon of immunosenescence in the elderly population significantly reduces immune surveillance function, thus creating conditions for the development of tumors, which is also one of the reasons why elderly people have a high incidence of hematological malignancies.

Recent research has revealed new mechanisms of immunosenescence and potential intervention strategies. The team of Jin Jun from Fudan University pointed out that T cell aging is the core of immunosenescence, and its intervention can help delay immunosenescence and reduce the occurrence of age-related diseases [179]. The Huanghe/Qian Pengxu team of ZJU released the first metabolic map of young and aging hematopoietic system, and found that uridine can restore the vitality of aging hematopoietic stem cells by up-regulating the FoxO signaling pathway to enhance self-renewal and inhibit inflammation [180]. The team of Makoto Nakanishi from the University of Tokyo found that the heterogeneous expression of PD-L1 in senescent cells plays an important role in the accumulation of senescent cells and age-related inflammation. Immune checkpoint blockade may be a promising anti-aging treatment strategy [181]. James Kirkland, MD, from the Mayo Clinic, first proposed the term "Senolytics", which specifically refers to drugs that selectively kill senescent cells. Drugs such as dasatinib, quercetin, and navitoclax have shown health benefits and extended lifespan in animal models [182]. In summary, adjusting a healthy lifestyle and appropriate anti-inflammatory strategies, including the use of specific anti-aging drugs, can effectively delay the aging process and may bring new hope for the prevention or treatment of chronic diseases related to age in the elderly. More new treatment methods will emerge to improve the survival situation of elderly patients.
